# Alarm Variables for Dengue Outbreaks: A Multi-Centre Study in Asia and Latin America

**DOI:** 10.1371/journal.pone.0157971

**Published:** 2016-06-27

**Authors:** Leigh R. Bowman, Gustavo S. Tejeda, Giovanini E. Coelho, Lokman H. Sulaiman, Balvinder S. Gill, Philip J. McCall, Piero L. Olliaro, Silvia R. Ranzinger, Luong C. Quang, Ronald S. Ramm, Axel Kroeger, Max G. Petzold

**Affiliations:** 1 Department of Vector Biology, Liverpool School of Tropical Medicine, Liverpool, United Kingdom; 2 Ministry of Health, Mexico City, Mexico; 3 Ministry of Health, Brasilia, Brazil; 4 Ministry of Health, Kuala Lumpur, Malaysia; 5 UNICEF/UNDP/World Bank/WHO Special Programme for Research and Training in Tropical Diseases (TDR), Geneva, Switzerland; 6 Institute of Public Health, University of Heidelberg, Heidelberg, Germany; 7 Pasteur Institute, Ho Chi Minh City, Vietnam; 8 Ministry of Health, Santo Domingo, Dominican Republic; 9 University of Gothenburg, Gothenburg, Sweden; China Medical University, TAIWAN

## Abstract

**Background:**

Worldwide, dengue is an unrelenting economic and health burden. Dengue outbreaks have become increasingly common, which place great strain on health infrastructure and services. Early warning models could allow health systems and vector control programmes to respond more cost-effectively and efficiently.

**Methodology/Principal Findings:**

The Shewhart method and Endemic Channel were used to identify alarm variables that may predict dengue outbreaks. Five country datasets were compiled by epidemiological week over the years 2007–2013. These data were split between the years 2007–2011 (historic period) and 2012–2013 (evaluation period). Associations between alarm/ outbreak variables were analysed using logistic regression during the historic period while alarm and outbreak signals were captured during the evaluation period. These signals were combined to form alarm/ outbreak periods, where 2 signals were equal to 1 period. Alarm periods were quantified and used to predict subsequent outbreak periods. Across Mexico and Dominican Republic, an increase in probable cases predicted outbreaks of hospitalised cases with sensitivities and positive predictive values (PPV) of 93%/ 83% and 97%/ 86% respectively, at a lag of 1–12 weeks. An increase in mean temperature ably predicted outbreaks of hospitalised cases in Mexico and Brazil, with sensitivities and PPVs of 79%/ 73% and 81%/ 46% respectively, also at a lag of 1–12 weeks. Mean age was predictive of hospitalised cases at sensitivities and PPVs of 72%/ 74% and 96%/ 45% in Mexico and Malaysia respectively, at a lag of 4–16 weeks.

**Conclusions/Significance:**

An increase in probable cases was predictive of outbreaks, while meteorological variables, particularly mean temperature, demonstrated predictive potential in some countries, but not all. While it is difficult to define uniform variables applicable in every country context, the use of probable cases and meteorological variables in tailored early warning systems could be used to highlight the occurrence of dengue outbreaks or indicate increased risk of dengue transmission.

## Introduction

Dengue epidemics have increased in frequency and magnitude globally since the 1970s [1], and today represent a major, and still increasing, public health burden worldwide [2]. The primary vector, *Aedes aegypti*, is highly anthropophilic [3] and breeds exclusively in small, freshwater bodies. It thrives in dense, urban areas where it has evolved to complete its entire life cycle [3]. A secondary vector, *Aedes albopictus* has expanded its range dramatically in recent decades, and is a serious threat for dengue transmission among populations where herd immunity is low or absent [4]. With all four dengue serotypes now found worldwide [5], outbreaks caused by a change in the predominant serotype form a major, global public health challenge [4].

Outbreaks can exert large pressures on public health systems, as hospitals and outpatient clinics become overwhelmed by the surge in true dengue positive cases, as well as other febrile illnesses [6,7]. These pressures are compounded by resource-limited or weak surveillance systems that might have given prior warning if sufficient funding, expertise and methodologies were in place [8–11]. Arguably, the ability to predict outbreaks with a generous lag time should enable public health systems to respond more efficiently through the timely allocation of resources [6,12,13]. It is in this capacity that infectious disease modelling has become increasingly relevant [12,14–16].

To date, epidemiological variables, such as the historic incident mean plus 2 standard deviations (SD), have been used to forecast dengue outbreaks with some success [17–20]. Regression functions are also a common feature of dengue modelling, and are used to calculate the probability of an outbreak, as reported recently in Vietnam [17] and Singapore [21]. These analyses identified clear trends between abnormal changes in meteorological and/ or epidemiological variables and subsequent dengue outbreaks.

Yet, due to the complex interactions between vector, pathogen and human [22], models struggle to accurately capture spatial and temporal data required to project the intricate transmission dynamics of dengue [23]. And while predictive models exist, these tend to focus on smaller spatial units, which are often inadequate for the district- or country-level responses required for public health control interventions [18–20,24]. Programme managers and regional epidemiologists alike need user-friendly, early warning systems (EWS) that can adequately explain inter-district dengue variation [13,25]. Novel approaches are required to develop predictive, accessible methodologies that utilise alarm variables on broad spatial scales [13]. To this end, we considered the Shewhart method and Endemic Channel to build a simple model based on logistic regression that can predict forthcoming outbreaks, with high sensitivity (number of true positive outbreak detections) and a low number of false alarms (PPV).

The Shewhart method is typically used to monitor the quality control of goods within the manufacturing process [26]. This method involves the use of control charts to define ‘in-control’ and ‘out-of-control’ manufacturing states, using the historic mean and standard deviation of the outcome variable [26,27]. Within a dataset, this method can identify variation that is beyond the influence of natural, random fluctuation, *i*.*e*. the consequence of an identifiable or ‘attributable’ cause or change in the process [14,27,28]. Since regional epidemiologists often collect historical data to calculate the moving incident mean (or median), applying this approach to infectious diseases modelling becomes possible.

These data can be used to forecast changes in the variable of interest, which is the primary basis of the Endemic Channel calculation [29]. In this sense, the Endemic Channel represents the number of cases within the expected normal range, or the ‘in control’ state, while anything above this moving threshold would be considered representative of an unprecedented number of cases and an ‘out of control’ state *i*.*e*. an outbreak. This approach is favoured in many countries, as it allows programme managers to easily define the presence/ absence of an outbreak [6,10,30], despite the limitations associated with abnormally high historic means and the variation in the seasonal timing of dengue cases [6]. Such predictive methodologies have demonstrated success in both Puerto Rico and Thailand [14,15,24], where measuring a prior increase in the outcome variable enabled models to retrospectively predict subsequent outbreak periods, thus indicating potential in prospective operational capacities. Extending this rationale further, it should be possible to investigate a preceding rise in meteorological, entomological and epidemiological independent variables, or alarm variables, to predict dengue outbreaks.

In spite of the progress made in modelling high risk areas and population dynamics [13,31–33], reliable, affordable and practical dengue early warning systems are still needed to mitigate the growing economic and human costs of dengue [25]. Accordingly, as part of IDAMS (International Research Consortium on Dengue Risk Assessment, Management and Surveillance) and the WHO-based Special Programme for Research and Training in Tropical Diseases (TDR), this paper describes the development and evaluation of an early warning system using the Shewhart method and Endemic Channel to predict dengue outbreaks at the district and country level in five countries in Asia and Latin America.

## Materials and Methods

### Objectives

Using retrospective country datasets, the aim was to define and detect dengue outbreaks using probable/ hospitalised cases as the outbreak variable [3], and successfully predict these outbreaks using earlier changes in various entomological, meteorological and epidemiological alarm variables.

### Data Collection

The five participating countries (Brazil, Dominican Republic, Mexico, Malaysia and Vietnam) were selected from a larger group whose dengue surveillance systems had been analysed previously [6,34]. A protocol for the data capture of a number of evidence-based alarm variables was agreed [6,10] and a data capture spreadsheet using Microsoft Excel was created. Participating countries conducted active data collection from October 2013 to April 2014. Data from 7 years (2007–2013) were collected and split into two periods: a 5-year historic period (2007–2011), used to calibrate and parameterise the model, and a 2-year evaluation period (2012–2013), used to test the model. WHO-TDR support staff periodically visited each country to ensure that data capture was completed accurately and to verify data sources to reduce the risk of misreporting. Each visit was documented and known problems were communicated.

All data were collected in-country with the cooperation of the Ministries of Health. The temporal unit was the epidemiological week (Sunday to Saturday) and the spatial unit was based on pre-existing political boundaries, most commonly the district (the municipality in Brazil; the locality in Mexico). The following variables were captured using the Excel spreadsheet:

- Meteorological (outdoor mean air temperature, rainfall, outdoor relative humidity);
- Epidemiological (mean age, circulating serotype, probable dengue cases [3], hospitalised dengue cases [3]);
- Entomological (Breteau Index, House Index, Ovitrap Index (Mexico only)).

Datasets were hugely variable but in general described an increase in temperatures and outbreak intensity, depending on the country context.

All meteorological data were matched to the district of interest to minimise spatial bias, although this was not possible in Malaysia. Paucity among meteorological datasets was sometimes high; consequently, external websites Wunderground [35] and Tutiempo [36] were used to augment the data collection. Where this was not possible, no meteorological variables were captured (Vietnam only). No remote sensing data were collected or used.

Weeks 1 and 53 for all variables were excluded due to inconsistent data quality. All patient medical data were anonymised. Microsoft Excel was used to transform daily data to weekly units and build epidemiological, entomological and meteorological datasets.

Due to paucity among datasets, logistic models for each district were not possible. Therefore, while meteorological data were not aggregated at the country level, one logistic model was based on data from all districts *i*.*e*. the same relationship observed at the national level between alarm variables and outbreaks was assumed to exist to the same degree within each district.

### The Endemic Channel

Two Endemic Channels were created using the outbreak variables probable cases and hospitalised cases. Each Endemic Channel was used in two prediction models to quantify outbreaks. Each Endemic Channel was defined and calculated as follows:

$$\frac{No.\;of\;weekly\;hospitalised\;cases}{District\;population}$$

$$\frac{No.\;of\;weekly\;probable\;cases}{District\;population}$$

The Endemic Channel was calculated for each district using a smoothed 13-week (6+1+6 week) moving mean and standard deviation, based on data in the historic period [14,28,37]. Using a multiplier of the standard deviation known as ‘z’, it was possible to vary the Endemic Channel within the evaluation period.

**Fig 1:**
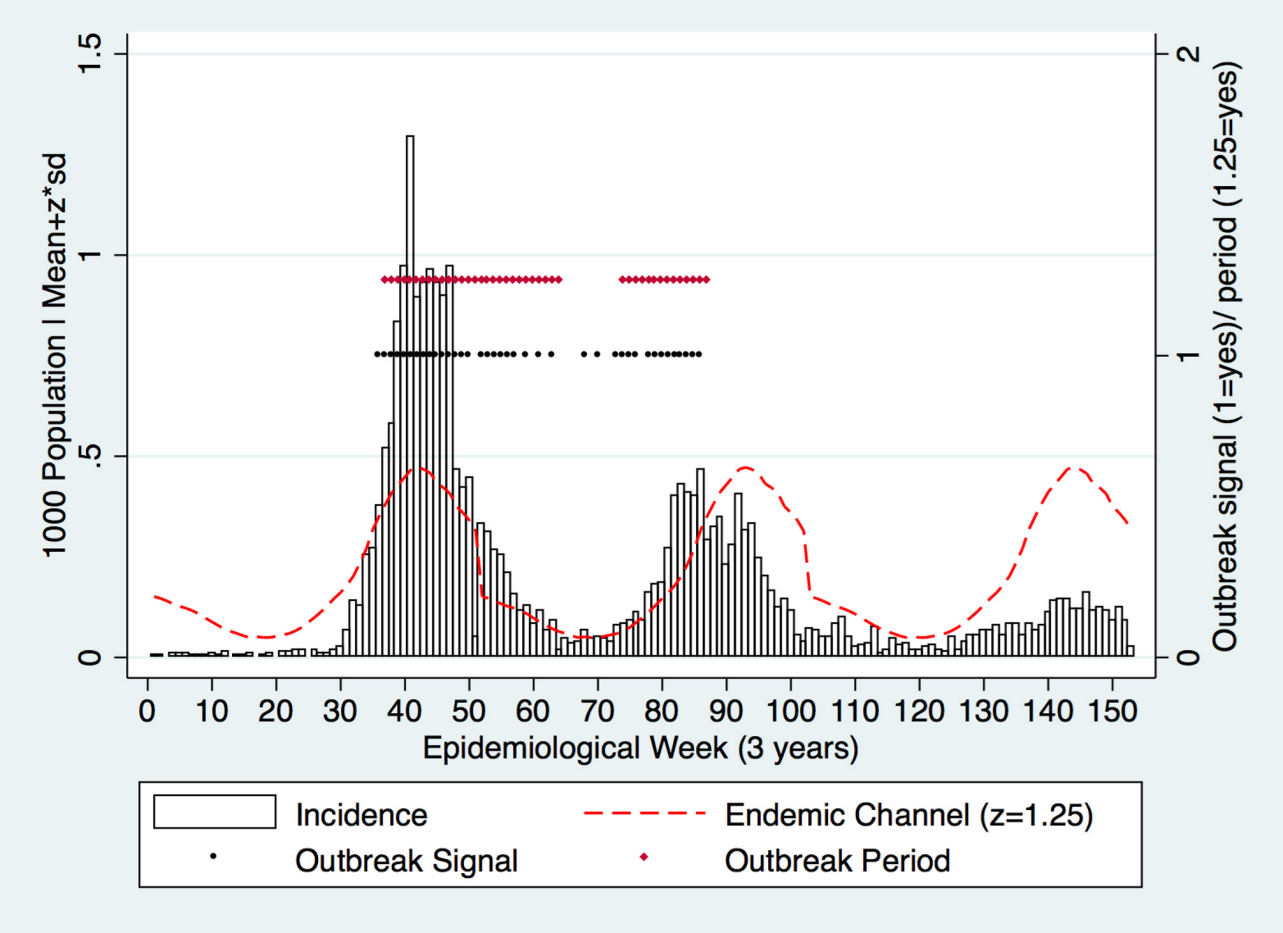
Modelling with a test dataset (3 years only) using the z value (z = 1.25) to form the Endemic Channel. Outbreak signals were detected (black dots) where incidence crossed the Endemic Channel. Outbreak periods (red dots) were formed when 2 consecutive outbreak signals were present; outbreak periods ended when 2 absent consecutive outbreak signals were registered (incidence did not cross the Endemic Channel for 2 consecutive weeks).

Incident cases with a value above the Endemic Channel triggered outbreak signals. Outbreak signals were combined into outbreak periods. An outbreak period was begun when 2 consecutive outbreak signals were detected; the same outbreak period ended when the outbreak signal had been absent for 2 consecutive weeks (Fig 1). Epidemic years were not excluded.

### The Shewhart Method

Alarm variables were used to detect outbreaks. Each variable is described below, including any formulae used.

1) Relative change in mean age of dengue incident cases was calculated using a smoothed average due to noisy, low frequency data. The formula used is as follows:

$$\frac{Smoothed\;average\;at\;week\;X-(smoothed\;value\;at\;week\;X-1)}{Smoothed\;value\;at\;week\;X-1}$$

2) Number of probable cases divided by the population (per 1,000 population)
3) Mean weekly outdoor temperature (weekly mean of daily means)
4) Total weekly rainfall
5) Mean weekly outdoor relative humidity (weekly mean of daily means)

### Model Calibration

Using data within the historic period, logistic regression was performed on the alarm variable (continuous) and outbreak signal (binary), which provided coefficients for use during the evaluation period. The statistical fit of the logistic regression model was not evaluated alone but considered as a part of the full outbreak detection model in terms of sensitivity and PPV. For each calendar week, data from all years were combined and separate models (in total 51 models from week 2 to 52) were estimated to accommodate for seasonal differences in the relation between the alarm variable levels and the risk of an outbreak. Accommodating these temporal patterns by simply estimating one model per calendar week implies that data from all districts need to be used and limits the possibilities for a spatial breakdown of the analysis. Hence, each observed relationship between alarm variable and outbreak signal was assumed to exist on a countrywide-basis, even though there were likely differences at smaller spatial scales.

Subsequently, each coefficient, together with the absolute value for the alarm variable in the current week, was used to calculate the outbreak probability during the evaluation period. This outbreak probability was plotted on a weekly basis against an artificial threshold, known as the alarm threshold. An alarm signal was triggered when the outbreak probability crossed the alarm threshold.

To reduce spurious associations with outbreak periods, weekly alarm signals were combined to form alarm periods, which were equal to 2 alarm signals within the lag period (see definition below). Thereafter the alarm threshold was systematically altered between values of 0.08–0.2 to find a balanced environment within which alarms periods were formed. These alarm periods were used to predict outbreak periods, and as the basis for model performance outputs.

### Model Validation

The parameter settings, such as the threshold levels for outbreak and alarm and the definition of an outbreak period (2 outbreak signals vs. 3 outbreaks signals), were changed for each run.

#### Sensitivity

The successful detection of outbreaks was reported using sensitivity. The number of positive events *e*.*g*. alarms periods and outbreak periods, were used to calculate sensitivity by the following formula:

$$\frac{Outbreak\;periods\;detected\;by\;alarm\;periods}{Total\;no.\;outbreak\;periods}$$

#### Specificity

The number of negative events was not recorded due to the difficulty of defining absent alarm and outbreak periods.

#### Positive Predictive Value

The proportion of false positive alarms was calculated using the positive predictive value (PPV). The formula can be seen below. NB: Multiple positive events (alarm periods) were defined as correct, even if they were positive for the same outbreak.

$$\frac{No.\;of\;correct\;alarm\;periods}{Total\;no.\;of\;defined\;alarm\;periods}$$

#### Negative Predictive Value

The number of negative events was not recorded due to the difficulty of defining absent alarm and outbreak periods.

### Lag Period

Research has shown diverse effects of a range of lag times between independent variables and epidemic dengue transmission [38–41], however as yet no systematic review exists that can provide a definitive range of an appropriate lag time for each covariate. There is evidence that early monitoring and targeting of both the ‘quiet phase’, when cases are few in the inter-epidemic period, and the ‘development phase’, characterised by increasing number of cases, can provide the most effective and timely period in which to intervene [22]. Accordingly, evidence from these sources was discussed in detail in consultation with expert opinion [42] before defining each lag period, detailed below:

- Temperature: 1–12 weeks before the outbreak
- Rainfall: 3–12 weeks before the outbreak
- Humidity: 2–12 weeks before the outbreak
- Mean Age: 4–16 weeks before the outbreak
- Breteau Index: 1–8 weeks before the outbreak
- House Index: 1–8 weeks before the outbreak
- Ovitrap Index: 2–8 weeks before the outbreak
- Probable Cases: 1–4 weeks before the outbreak (altered to 1–12 weeks due to too few alarm periods)

### Data Analysis

Analyses were run in duplicate, independently by two of the authors (LRB and MP) to limit systematic error. The Endemic Channel and Shewhart method were programmed in Stata 13.1 [43].

### Proof of Concept

As a starting point, multiple runs with a test dataset were conducted to analyse the reliability of the model and consistency of the approach. A test dataset is defined as a dataset that is 100% complete and reliable that was taken from multiple sources to act as a ‘control’ for the model. It was necessary to evaluate the model in this capacity to generate results and demonstrate proof of concept *i*.*e*. when datasets are complete, accurate and reliable, this is the way in which the early warning system uses and interprets the data.

### Alarm and Outbreak Thresholds

**Fig 2:**
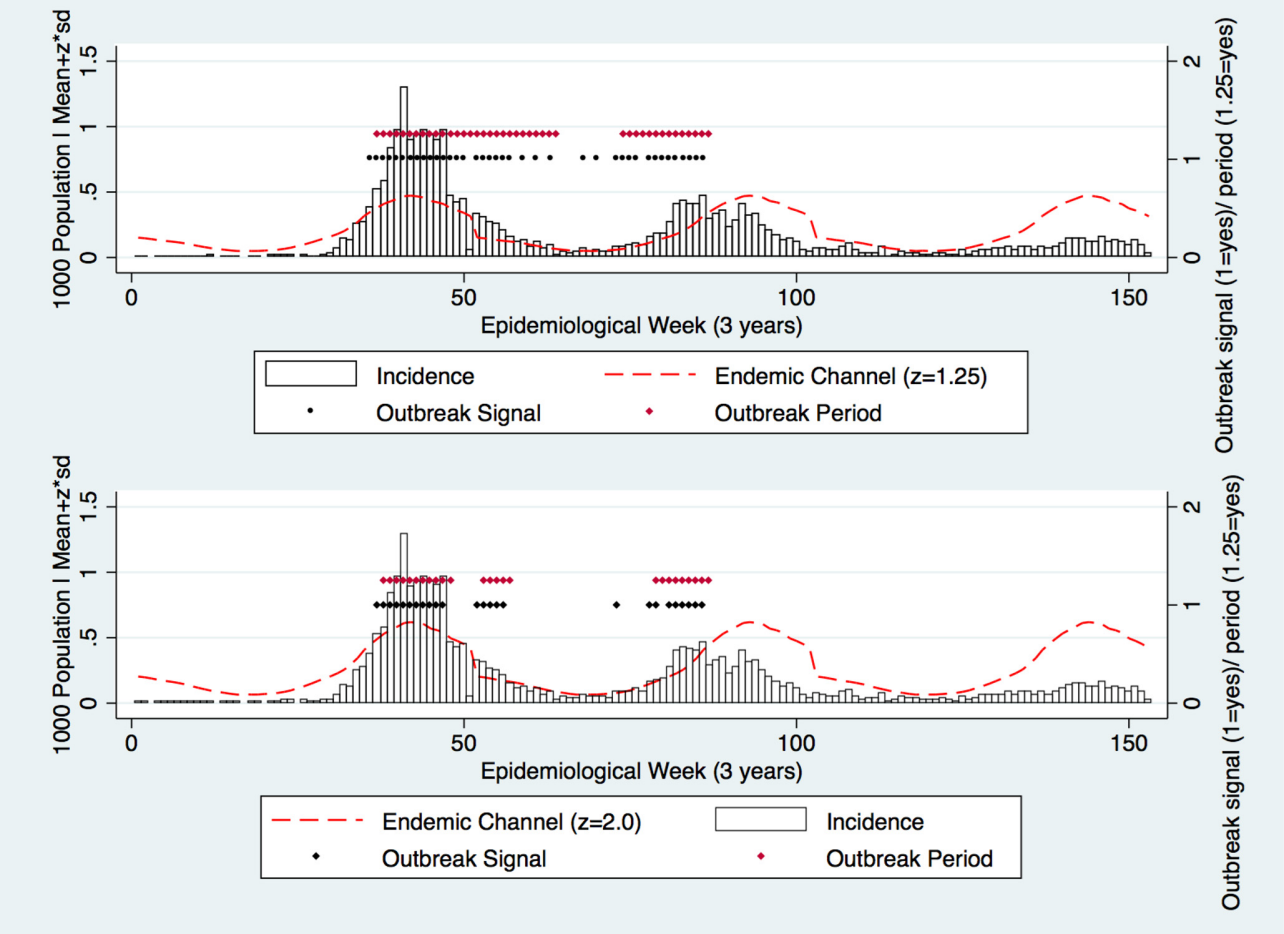
Modelling with a test dataset (3 years only) using two z values (Top: z = 1.25; Bottom: z = 2.0) to form the Endemic Channel. Outbreak periods (red dots) were equal to two consecutive outbreak signals (black dots) and ended in the absence of 2 consecutive outbreak signals.

Altering the z-value was the only method used to change the Endemic Channel and generate outbreak periods. As z was increased, fewer outbreak periods were generated (Fig 2). At a low z-value, outbreak periods were generated by relatively low magnitude incidence, and were continually recorded for long durations (Fig 2). Thereafter, as the z-value increased, lower magnitude incidence did not cross the Endemic Channel, and the number of outbreak periods became less frequent.

**Fig 3:**
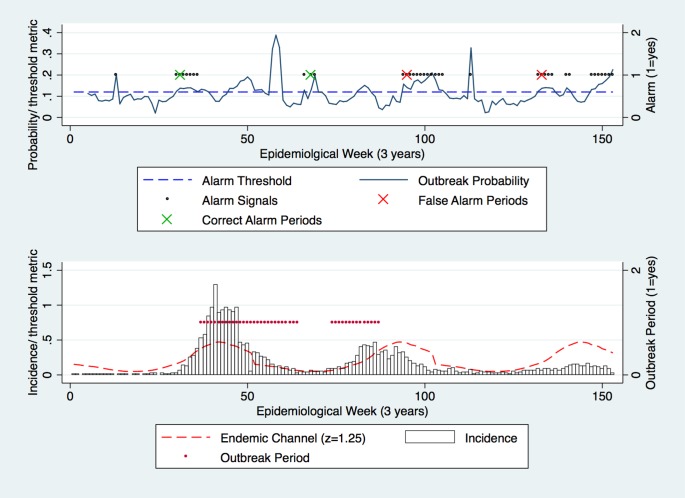
Test dataset: Alarm threshold of 0.12 was used against the outbreak probability. Alarm periods (defined by 2 alarm signals (black dots) within the lag period) successfully detected outbreak periods (red dots) (defined by 2 outbreak signals). Correct and false alarms are highlighted. z = 1.25.

**Fig 4:**
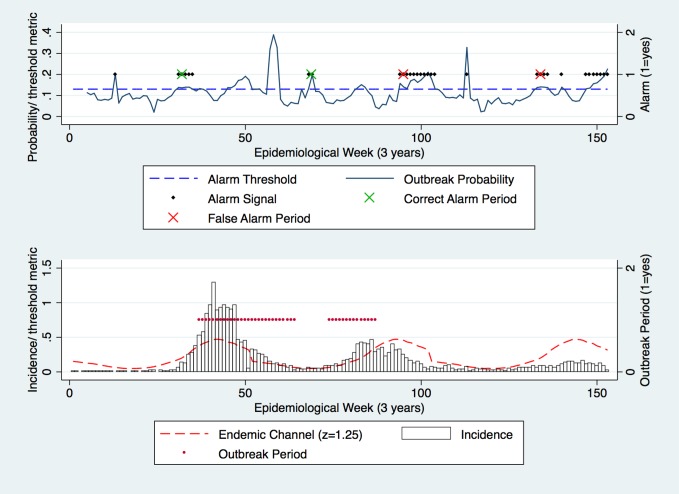
Test dataset: Alarm threshold of 0.13 was used against the outbreak probability. Alarm periods (defined by 2 alarm signals (black dots) within the lag period) successfully detected outbreak periods (red dots) (defined by 2 outbreak signals). Correct and false alarms are highlighted. z = 1.25.

In a similarly systematic approach, the outbreak probability was tested against the alarm threshold to generate alarm signals/ periods prior to outbreak periods (Fig 3). As with the Endemic Channel and outbreak periods, alarm period frequency also decreased as the alarm threshold was increased (Figs 3 and 4).

### Alarm and Outbreak Definitions

**Fig 5:**
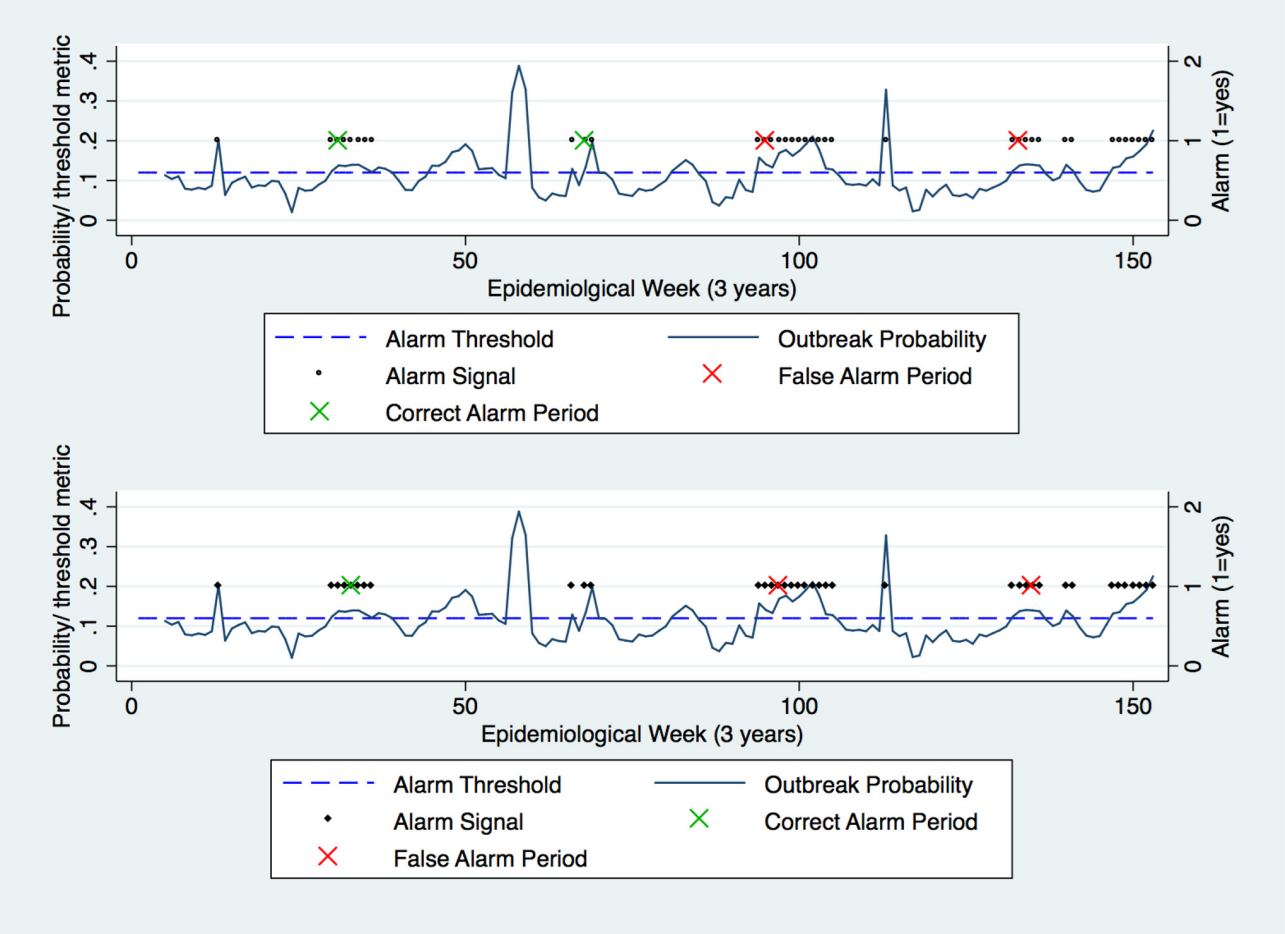
Test dataset: Alarm threshold = 0.12, z-value = 1.25. Top: alarm periods defined by 2 alarms signals (black dots). Bottom: alarm periods defined by 4 alarm signals.

To ensure that detection times were reasonably short, 2, 3 and 4 weekly alarm/ outbreak signals were used to define alarm/ outbreak periods. Altering the number of signals required to form an alarm/ outbreak period increased or decreased the frequency of alarm/ outbreak periods (Fig 5). It also affected the temporal relationship between alarm and outbreak periods by altering the week at which alarm/ outbreak periods were observed (Fig 5). In prospective terms, increasing the number of alarm signals required to form outbreak periods delayed detection times. In addition, the analyses showed that using 2 or 3 alarm/ outbreak signals to form alarm/ outbreak periods generated highest model performance metrics. Considering these results, the model was parameterised using 2 signals as this reduced detection delay and resulted in higher model performance.

### Ethical Permission

Ethical approval for the study protocol was sought from and granted by WHO Regional Ethical Committees, specifically the Pan American Health Organization Ethics Review Committee (PAHO-ERC; Ref No. 2011-12-0021) and the Western Pacific Regional Office Ethics Review Committee (WPRO-ERC; Ref. 2013.25.ICP.2.ESR). All patient medical data were anonymised.

## Results

### Model Performance Evaluation

After demonstrating the functionality of the model using a test dataset (Figs 1–5), country datasets (evaluation period) were subsequently used.

**Fig 6:**
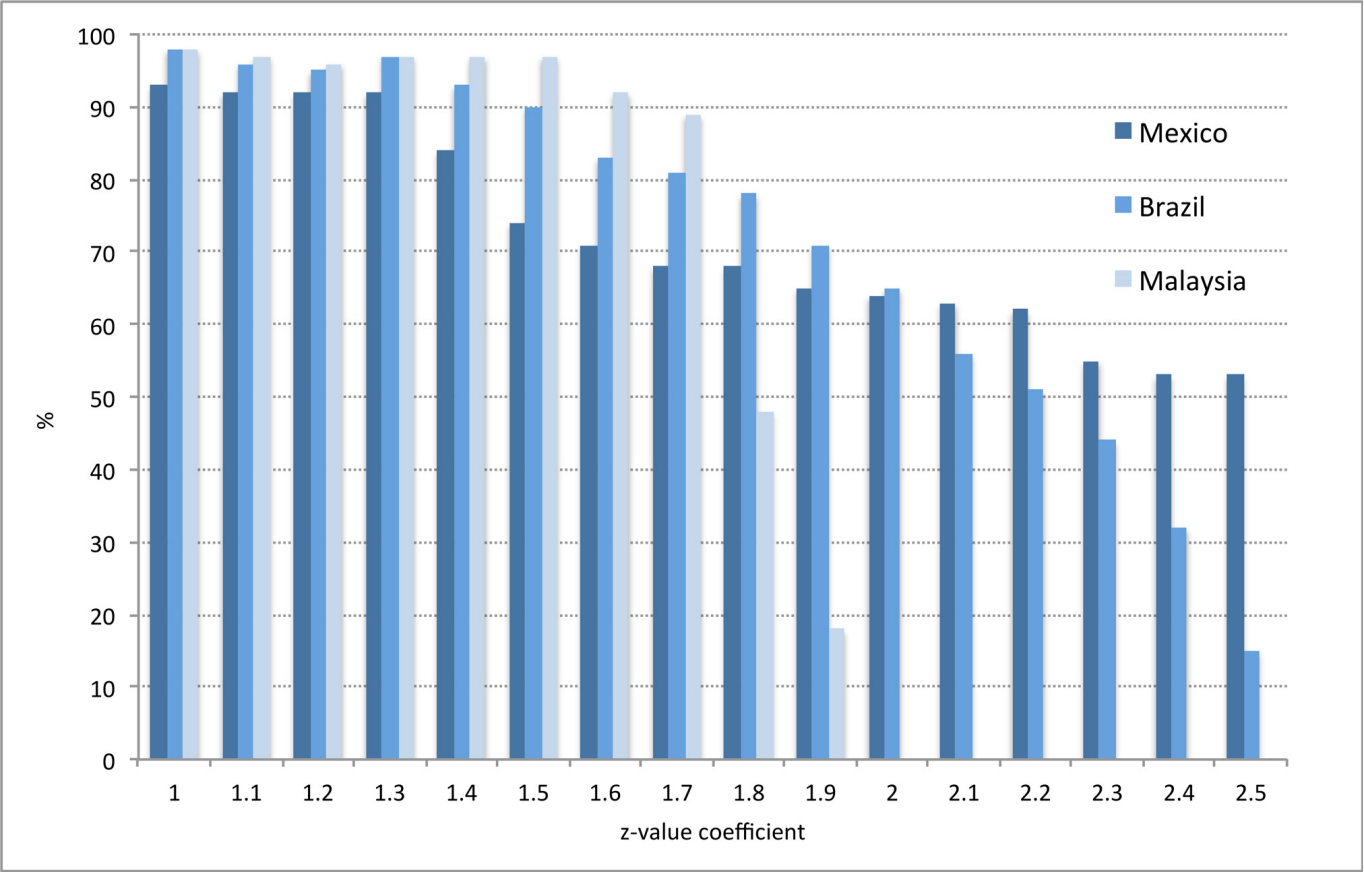
Performance testing of the outbreak probability using 3 country datasets (evaluation period). Sensitivity. z-value = 1.25, alternative alarm thresholds. Brazil: Alarm variable = Probable Cases; Outbreak variable = Hospitalised Cases; Mexico: Alarm variable = mean temperature; Outbreak variable = Hospitalised Cases; Malaysia: Alarm variable = Mean age; Outbreak variable = Hospitalised Cases.

**Fig 7:**
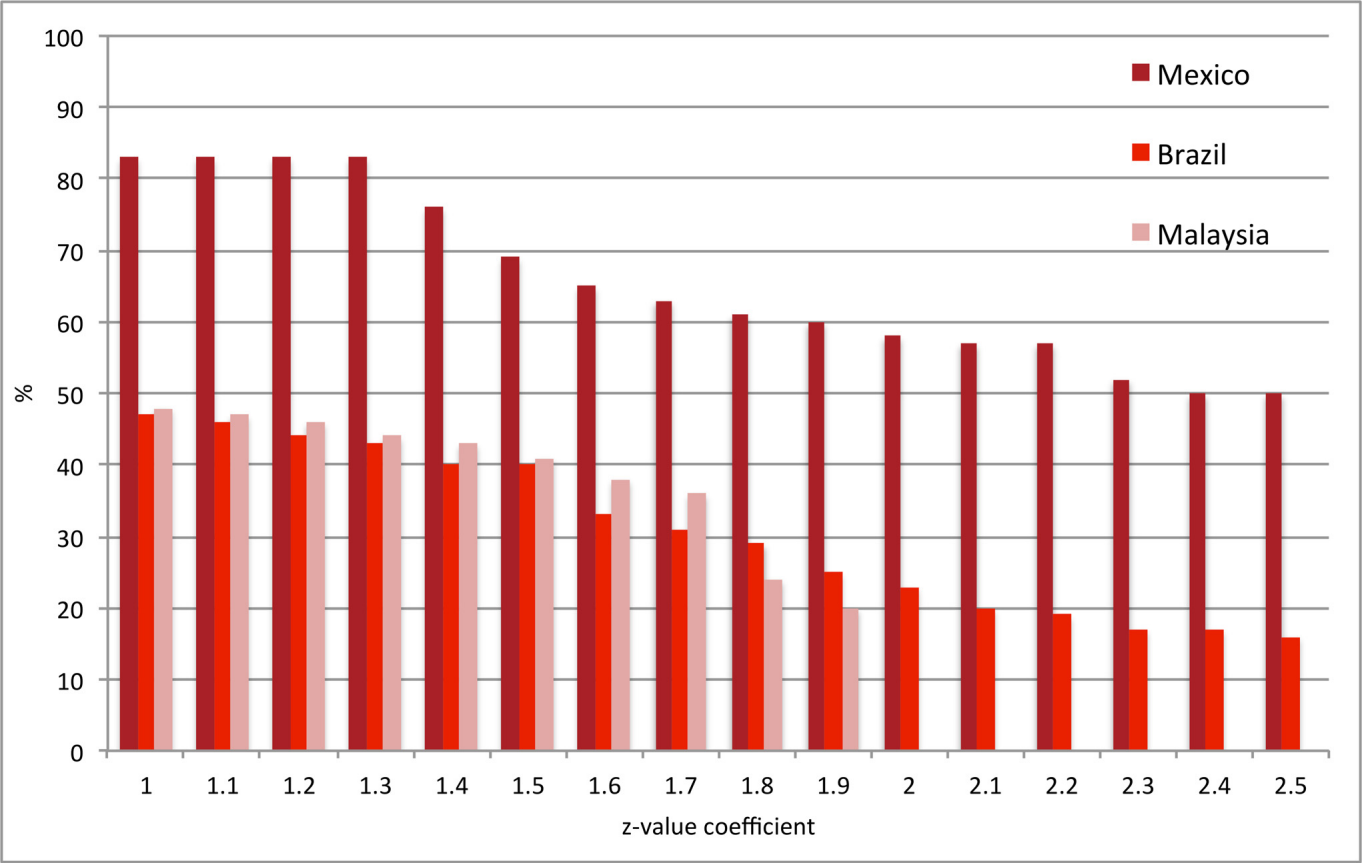
Performance testing of the outbreak probability using 3 country datasets (evaluation period). Positive Predictive Value. z-value = 1.25, alternative alarm thresholds. Brazil: Alarm variable = Probable Cases; Outbreak variable = Hospitalised Cases; Mexico: Alarm variable = Mean Temperature; Outbreak variable = Hospitalised Cases; Malaysia: Alarm variable = Mean Age; Outbreak variable = Hospitalised Cases.

**Fig 8:**
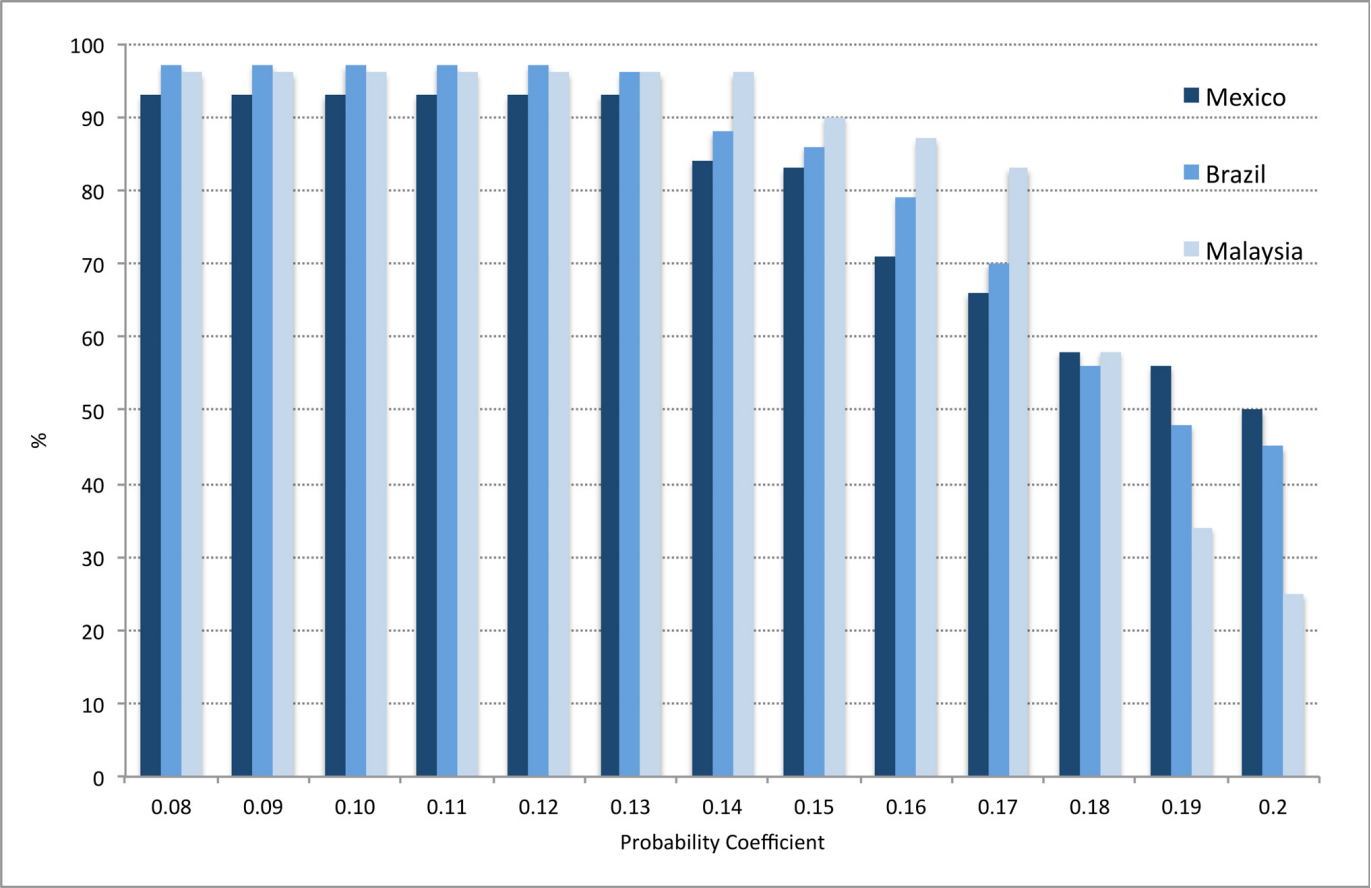
Performance testing of the outbreak probability using 3 country datasets (evaluation period). Sensitivity. Alarm threshold = 0.12, alternative z-values. Brazil: Alarm variable = Probable Cases; Outbreak variable = Hospitalised Cases; Mexico: Alarm variable = Mean Temperature; Outbreak variable = Hospitalised Cases; Malaysia: Alarm variable = Mean Age; Outbreak variable = Hospitalised Cases.

**Fig 9:**
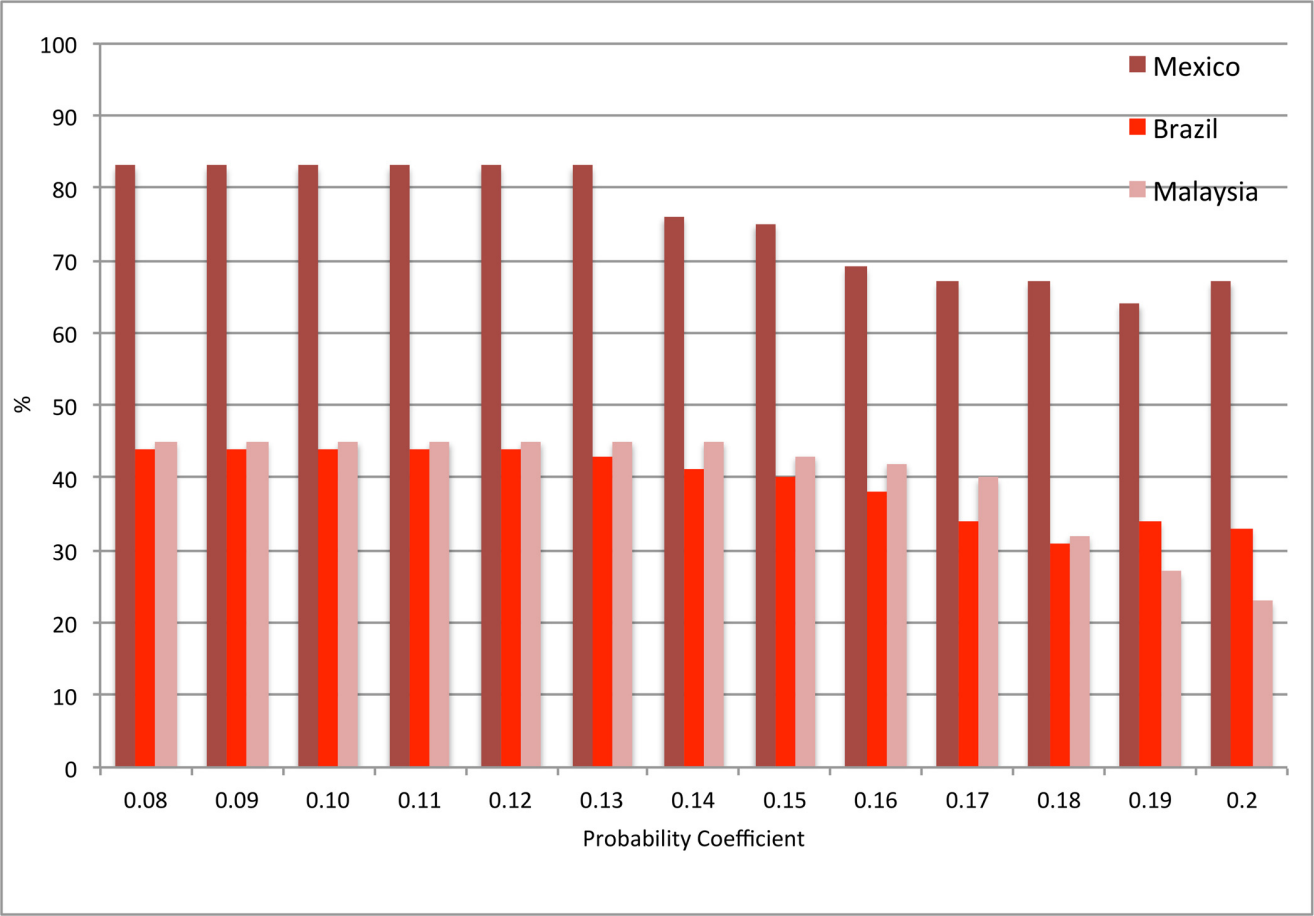
Performance testing of the outbreak probability using 3 country datasets (evaluation period). Positive Predictive Value. Alarm threshold = 0.12, alternative z-values. Brazil: Alarm variable = Probable Cases; Outbreak variable = Hospitalised Cases; Mexico: Alarm variable = Mean Temperature; Outbreak variable = Hospitalised Cases; Malaysia: Alarm variable = Mean Age; Outbreak variable = Hospitalised Cases.

Firstly, z-values and outbreak probabilities that provided sufficient alarms and outbreaks were determined. A systematic approach ensured that all z and alarm threshold values were tested incrementally, using three of the five country datasets (Brazil, Mexico, Malaysia), as these were most complete at this stage. Results indicated that, despite altering the alarm and outcome variables, a z-value of between 1.0–1.3, and an alarm threshold of between 0.08 and 0.12, yielded the best model performance (Figs 6–9). Higher coefficients of either outbreak probabilities or z-values resulted in marked decreases in sensitivity, and to some extent, PPV (Figs 6–9). Hence, z = 1.25 and an alarm threshold = 0.12 as parameters for all subsequent evaluations of country datasets. These country results can be seen in Table 1.

**Table 1:** Summary results table stratified by country (data from evaluation period). Most sensitive variables stratified by country where z = 1.25 and probability = 0.12.

Country	Alarm Variable	Outbreak variable	Lag Period (weeks)	Sensitivity (%)	Positive Predictive Value (%)
Mexico	Mean Temperature	Hospitalised Cases	1–12	79	73
Mexico	Rainfall	Hospitalised Cases	3–12	59	63
Mexico	Mean Age	Hospitalised Cases	4–16	72	74
Mexico	Probable Cases	Hospitalised Cases	1–12	93	83
Brazil	Mean Temperature	Hospitalised Cases	1–12	81	46
Brazil	Probable Cases	Hospitalised Cases	1–12	97	43
Brazil	Rainfall	Hospitalised Cases	3–12	70	33
Brazil	Mean Humidity	Hospitalised Cases	2–12	79	46
Brazil	Mean Temperature	Probable Cases	1–12	49	50
Brazil	Mean Age	Hospitalised Cases	4–16	86	41
Malaysia	Mean Age	Hospitalised Cases	4–16	96	45
Malaysia	Mean Temperature	Hospitalised Cases	1–12	14	35
Malaysia	Mean Humidity	Hospitalised Cases	2–12	9	32
Dominican Republic	Rainfall	Hospitalised Cases	3–12	17	76
Dominican Republic	Mean Temperature	Hospitalised Cases	1–12	24	82
Dominican Republic	Mean Humidity	Hospitalised Cases	2–12	6	80
Dominican Republic	Probable Cases	Hospitalised Cases	1–12	97	86
Dominican Republic	Mean Humidity	Probable Cases	2–12	5	71
Dominican Republic	Mean Temperature	Probable Cases	1–12	23	81
Dominican Republic	Rainfall	Probable Cases	3–12	16	70
Vietnam	Mean Age	Probable Cases	4–16	57	45
Vietnam	Probable Cases	Hospitalised Cases	1–12	93	43

## Discussion

Early warning systems are becoming more important as a tool to mitigate the impact of disease outbreaks [44]. Clearly, alarm variables that provide advance warning of outbreaks are the most valuable, in order to enact timely clinical preparations and vector control responses. It is crucial that these same alarm variables should not trigger too many false positive alarms, as this would result in reduced confidence in the EWS, primarily due to wasted resources. In this study, the Shewhart method and Endemic Channel were used to evaluate alarm variables with the potential to predict subsequent outbreak periods. A number of epidemiological and meteorological variables were tested, primarily to evaluate their predictive potential, and secondarily to establish the most appropriate case definition to define outbreaks.

Surprisingly, despite inherent variability throughout the datasets, certain meteorological and epidemiological alarm variables were predictive across all countries. These findings are consistent with trends and evidence reported elsewhere at smaller spatial levels [17–21,45].

### Epidemiological Variables

#### Alarm variable: probable cases

Epidemiological variables have already been used to predict outbreaks retrospectively with some success [45]. Of the epidemiological alarm variables studied here, probable dengue cases demonstrated the greatest predictive capacity. In Mexico and the Dominican Republic, sensitivity and PPV were high, at 93%/ 83% and 97%/ 86% respectively, while in Brazil and Vietnam, model performance was 97%/ 43% and 93%/ 43% respectively (Table 1) (No data were available from Malaysia). From these data it is clear that the use of probable cases as an alarm variable was highly sensitive across all countries, and while the same broad success cannot be said for PPV, still in Mexico and Dominican Republic, this output was relatively high–high enough to ensure few false alarms in practice. The metrics demonstrate that models that work well in one country context can also be beneficial in others.

In both Dominican Republic and Mexico, it could be argued that the success achieved was because incidence during the period of evaluation better reflected the patterns observed during the historic period, *i*.*e*. that outbreaks were aligned in time and space throughout the historic and evaluation periods, thereby resulting in fewer false alarms. While on the contrary, in Brazil and Vietnam, the lower PPV values could be attributed to noisier datasets that were inconsistent across the years. This would not be surprising given that the length of historic periods was relatively low when compared to similar forecasting models [15,19,46].

There are almost certainly other factors at play here. The observed differences between PPV values could reflect the context-dependent nature of dengue transmission, which has long been argued as a feature of dengue [47–49]. Equally, country surveillance systems are often unique, resulting in heterogeneous case registration and reporting systems [10,30]. Indeed these differences could also be due to the presence of co-circulating infections with similar clinical presentations, such as Chikungunya or Zika viruses [50,51], which may be confounding probable dengue case diagnoses, or because case definitions are less specific (or likely a combination of both) [4,52].

Nonetheless, while suspected or probable cases are notifiable within many existing disease surveillance systems [10,30], these data suggest that probable case data can, in some cases, be predictive of dengue outbreaks and should be considered for use in early warning systems.

#### Alarm variable: mean age

Theoretically, since population-level serotype shifts are known to fluctuate inter-annually [53–56] thereby influencing the herd immunity of a population [55], it should be possible to detect such changes through a proxy increase or decrease in the mean age of infection [57]. Throughout these analyses the model performance metrics for mean age were extremely varied–the range of sensitivity was 57%–96% while PPV range was 41%-74%. Indeed, in the context of this specific model, these results indicate that the use of a change in mean age may warrant further investigation, but due to inconsistency, mean age can not currently be recommended for use in early warning systems.

The limited success of this model in using mean age as a predictor for dengue outbreaks may in part be due to the following limitations. Firstly, it was not possible to correlate the incident age distribution of dengue with serotype shifts to stratify the risk of infection among age groups, primarily due to inconsistent data entry. Secondly, mean age was calculated as either a function of probable or hospitalised cases, perhaps masking true associations that may have been more pronounced if the calculation had been standardised across countries. Finally, where the calculation of mean age was based on probable cases, the effect of poorer specificity within this case definition likely diluted any associations with the outbreak variable probable cases, which may explain why all countries, excepting Vietnam, generated lower PPV values when compared with the outbreak variable hospitalised cases.

#### Meteorological variables

Countries that had access to better meteorological datasets (Mexico and Brazil) produced higher performance metrics when compared to those countries that captured fewer data points (Table 1). These were often spread disparately over wide geographic areas (Dominican Republic and Malaysia). It is likely that meteorological data captured over broader spatial scales poorly reflected the weather variation present over finer scales. Consequently, any outbreak probability calculated using these data may not have been representative of the interactions between meteorological and outbreak variables in the district. Additionally, inconsistencies between the location of data captured for meteorological and outbreak variables within districts may have increased variability.

However, mean temperature was a reliable variable in Mexico, and to some extent in Brazil, where sensitivity and PPV were 79%/ 73% and 81%/ 46% respectively. Rainfall and humidity were more variable and generally less reliable early warning variables for dengue outbreaks. This was the case across all countries, but that is not to say that these variables should be ruled out of early warning systems altogether. It is certainly conceivable that each meteorological variable could indicate increased risk of transmission, rather than forecast an outbreak. Indeed, given their direct influence on vector population dynamics and on the extrinsic incubation period of the dengue virus, it is perhaps not surprising that meteorological variables have demonstrated potential, both in this study and in various mathematical models also using field data [17,18,25].

That mean temperature generally outperformed both rainfall and humidity is unsurprising. Associations between temperature and dengue have been observed before, as with other vector borne diseases, often as a consequence of the effect on the development rate of the vector and the extrinsic incubation period of the pathogen [58–61]. In particular, temperature variations are known to influence DENV replication, vector survival and larval development [13,62–64], while rainfall, or lack thereof, can affect the quantity and/ or quality of breeding sites [18,22,24,65,66]. Certainly, some of the variation observed within this study might be attributable to land use, vegetation, altitude and indeed human behaviour [67,68]—data that were not readily available during the data capture process. At the same time, spatial smoothing effects might also be a contributory factor, as district sizes were not standardised between countries—working at coarser resolutions tends to obscure or weaken associations often present at finer spatial scales. Nevertheless, the differences between countries in this study, particularly with regard to rainfall and humidity, are similar to other research that has also reported context-dependent meteorological alarm variables [69]. For example, rainfall has been positively associated with subsequent dengue outbreaks in a number of studies [70,71]. Indeed in Mexico, sensitivity and PPV was modest at 59%/ 63%, and although it has not been strongly predictive in all locations, there is the suggestion that all meteorological variables can play a part in the prediction of dengue outbreaks.

### Defining Outbreaks

#### Probable and hospitalised cases

Defining outbreaks using incident hospitalised cases, a common practice today [6], broadly demonstrated significantly better predictions with alarm variables when compared with incident probable cases. It is reasonable to presume that lower sensitivities and PPVs were likely the result of less specific dengue case definitions. If so, outbreak probabilities calculated during the historic period would have been consistently weaker, thereby reducing the performance metrics for each dataset accordingly. And yet, the utility of probable case definitions as outbreak variables should not be diminished. Similar trends observed between alarm variables and hospitalised cases were also seen between alarm variables and probable cases. Thus, this variable could be used as a substitute when developing Endemic Channels and epidemic curves, if timely reporting of hospitalised cases is not available [4].

#### The Endemic Channel

Worldwide, the multiplier ‘2’ is used to build the Endemic Channel using the following formula: mean+2*SD. This multiplier is used as it broadly captures 95% of the variation in dengue incidence about the mean. However, for the purposes of outbreak detection, this does not account for any localised variation that may warrant context-specific multipliers [47,72]. It is also important to identify that dengue incidence fluctuates on an inter-annual basis, and that the pattern of outbreaks may shift in time, frequency and duration [47]. Indeed, in terms of prediction, it is crucial to capture local covariates in order to anticipate whether the seasonal pattern of outbreaks is likely to change, perhaps due the presence of a new serotype early in the season [72,73]. In this model, it was not possible to capture such variation due to paucity among the datasets; hence regression coefficients were derived on a countrywide scale, arguably too coarse to detect such nuances.

So how should dengue outbreaks be defined? In this study, we altered z-values to improve the success of detection, rather than consider the operational or financial implications of changing outbreak definitions. These neglected implications had ramifications: low z-values resulted in outbreaks that were often infrequent, long and protracted in nature, and would require resource-intensive responses. We observed that as the z-value gradually increased, only the highest magnitude peaks were captured—it was even possible to create additional outbreaks as the z increased further (Fig 2). From these analyses it is clear that standardised thresholds failed to distinguish between certain types or stages of the outbreak.

Dengue transmission is often characterised by a series of peaks in incident cases, which is a function of variable intrinsic and extrinsic incubation periods [74]. The implication is that as one increases the z-value, there will come a point at which a greater frequency of distinct outbreaks is recorded, resulting in shorter duration but greater frequency outbreak responses (Fig 2). Consideration to the type of outbreak detected is rarely given, which would otherwise be beneficial to those in operational capacities. Indeed if it was possible to predict certain stages of an outbreak, such dichotomies (outbreak/ no outbreak) that arise from use of the Endemic Channel, would disappear. With it would go the mistrust that follows perceived unreliable or confusing forecasting [75]. So rather than focus on a simple binary output, perhaps it would be prudent to characterise outbreaks by a relative weekly increase in incidence, or indeed use the slope of the curve to forecast the top of the epicurve. Such a system would provide programme managers and epidemiologists with a more detailed insight into the speed and magnitude of future outbreaks, which would increase the efficiency and cost-effectiveness of dengue outbreak responses.

While the above techniques are under consideration, the results from this study suggest that the Endemic Channel meanwhile remains an operationally useful aid, primarily because of its ability to clearly demarcate thresholds based on simple summary statistics.

### Temporal Associations between Alarm and Outbreak Variables

#### Timely outbreak detection

Using 2 or 3 alarm/ outbreak signals to define alarm/ outbreak periods produced the highest outcome metrics, while there was little difference between these two multipliers across all alarm/ outbreak variables. As demonstrated previously, altering this multiplier can increase or decrease outbreak detection times (Fig 5), which is particularly important in a prospective context. Similarly, working with a moving average tends to delay the anticipated outbreak pattern by delaying the increase and postponing the decrease in incidence. In this study, the smoothing took place over 13 weeks (6+1+6), but this could be reduced to better reflect real-time events. However, this would be at the expense of increasing the impact of any noise in the dataset, also an important consideration prospectively.

### Candidate Alarm Variables

In addition to the alarm variables explored within this study, there is increasing evidence that novel variables may prove valuable in forecasting dengue outbreaks. Internet-based trending metrics can warn of forthcoming outbreaks, with evidence suggesting that these data might be useful for predicting dramatic surges in dengue incidence [8]. Both search query data [76,77] and social media trends [78] have shown promise for detection of disease outbreaks, although social media has yet to be evaluated for dengue. Other avenues of exploration could also include the use of alternative summary statistics for those alarm and outbreak variables already explored within this study, such as the diurnal temperature range instead of the mean temperature, or cumulative mean instead of the moving mean [47, 79]. And as the use of GIS-based and remotely sensed data capture becomes increasingly prevalent, spatial analyses and prediction based on the clustering nature of dengue, as well as geo-referencing of alarm variables, should enable scientists to better pinpoint potential high risk transmission areas at smaller spatial scales [46, 80–82].

### Limitations

Inconsistent data collection and missing data almost certainly affected the quality of datasets, especially with regard to entomological indices. As observed in another review [82], entomological indices were generated on varied temporal and/ or spatial scales in different countries, resulting in a mismatch with the outbreak variables. Accordingly, these alarm variables could not be fairly evaluated.

The following additional limitations in the routine surveillance data were observed:

- Temporal variation (monthly timescale observed for some entomological variables)
- Spatial variation (data, especially meteorological, were sometimes only available at coarser resolutions)
- Paucity/ absence of data/ variables
- Varied data sources (independent online systems)
- Multiple non-verifiable data sources
- Random (inconsistent) sampling (particularly entomological indices)
- Annual data entered only on one date rather than each week of the year
- As indicated above, mean age calculations were inconsistent between countries

The moving average and regression probabilities calculated during the historic period were reliant upon a relatively low number of years (<5) of historic data, in contrast to others forecasting models [15,18,46]. Using a greater number of historic years would have generated a more stable mean and outbreak probabilities.

Outbreak probabilities for alarm variables were based on countrywide associations, a spatial scale that smoothens variation found at the district level, potentially underestimating true probabilities. Also, due to co-linearity between variables, multivariate analyses were not conducted.

Generally, z-values of 1.25 and alarm thresholds of 0.12 were the most appropriate to generate high sensitivities and PPVs by country. The reason the z value is lower than the normal ‘2’, is due to the inclusion of epidemic years among the data, which if excluded would have necessitated larger standard deviations to detect outbreaks [6]. This combination of z value and alarm threshold would likely benefit from minor alterations to suit individual spatial units in any future prospective investigations.

Some variables, in particular temperature, have been known to show non-monotonic relations concerning mosquito and viral replication [69,63,83], however these effects were not captured in the current model.

## Conclusions

The findings reported here suggest that the Shewhart method and Endemic Channel,—relatively simple approaches—are viable techniques that can be used retrospectively, and potentially prospectively, to detect dengue outbreaks using alarm variables with an attributed lag time. This approach builds on earlier observations that utilised multiple alarm variables on similar spatial/ temporal scales [13], and combined prior theoretical observations into a practical model [25]. While there is emerging evidence of alternative models that may be used for time series datasets, in particular, the LASSO (least absolute shrinkage and selection) method [84], evidence suggests that such alternatives may require particularly complete and detailed datasets. Datasets compiled by mandatory electronic reporting and standardised surveillance systems will greatly improve the quality of datasets and lend themselves to such analyses. Until this point, simpler methods such as the Shewhart method and Endemic Channel may be more appropriate.

Of the epidemiological alarm variables studied, the number of probable cases showed greatest predictive potential and should be routinely captured during active surveillance systems for use in early warning systems. Increases in this metric may provide advance warning of increasing dengue outbreaks in subsequent time periods (in this study, 1–12 weeks). By contrast, the mean age of dengue cases requires further validation as a potential variable.

Meteorological alarm variables were more powerful predictors of outbreak periods in both Mexico and Brazil than other countries, likely due to more frequent spatial data points and accurate spatial correlations with outbreak variables. Therefore, where spatial meteorological data are discordant with the spatial area of analysis, interpolation or remote-sensing techniques should be used to generate additional climate data. Indeed, given the widespread availability of temperature and humidity data, dengue surveillance programmes should routinely record these metrics in order to detect any sustained, abnormal changes that may indicate increased risk of dengue transmission, as well as outbreaks.

Exploratory analyses of the value of entomological indices as predictors of epidemic dengue transmission are still required. This can only take place if study designs and data capture processes are standardised [85], thereby improving the quality of entomological datasets for use in predictive models.

In the absence of process-based models, predictive dengue modelling must be based on available retrospective datasets, validated across multiple contexts and parameterised for smaller spatial scales to capture local drivers in dengue transmission.

This model could be simply transformed into a real-time, user-friendly early warning system to identify at-risk areas in order to allocate resources more efficiently before outbreaks begin. At the time of writing, the model is deployed in a predictive capacity across 3 dengue-endemic countries, with initial results expected in the latter part of 2016.

## References

[1] Gubler DJ. The economic burden of dengue. Am J Trop Med Hyg. 2012;86: 743–744. 10.4269/ajtmh.2012.12-0157 22556068 PMC3335674

[2] Bhatt S, Gething PW, Brady OJ, Messina JP, Farlow AW, Moyes CL, et al The global distribution and burden of dengue. Nature. 2013;496: 504–507. 10.1038/nature12060 23563266 PMC3651993

[3] World Health Organization. DENGUE Guidelines for Diagnosis, Treatment, Prevention and Control. WHO 2009: 1–160. 23762963

[4] World Health Organization. Global Strategy for Dengue Prevention and Control 2012–2020. WHO 2012 8: 1–43.

[5] Messina JP, Brady OJ, Scott TW, Zou C, Pigott DM, Duda KA, et al Global spread of dengue virus types: mapping the 70 year history. Trends Microbiol. 2014;22: 138–146. 10.1016/j.tim.2013.12.011 24468533 PMC3946041

[6] Badurdeen S, Valladares DB, Farrar J, Gozzer E, Kroeger A, Kuswara N, et al Sharing experiences: towards an evidence based model of dengue surveillance and outbreak response in Latin America and Asia. BMC Public Health. 2013;13: 1–1. 10.1186/1471-2458-13-607 23800243 PMC3697990

[7] Simmons CP, Farrar JJ, Nguyen VVC, Wills B. Dengue. N Engl J Med. 2012;366: 1423–1432. 10.1056/NEJMra1110265 22494122

[8] Gluskin RT, Johansson MA, Santillana M, Brownstein JS. Evaluation of internet-based dengue query data: google dengue trends. PLoS Negl Trop Dis. 2014;8: e2713 10.1371/journal.pntd.0002713.t002 24587465 PMC3937307

[9] Madoff LC, Fisman DN, Kass-Hout T. A new approach to monitoring dengue activity. PLoS Negl Trop Dis. 2011;5: e1215 10.1371/journal.pntd.0001215.g001 21647309 PMC3104030

[10] Runge-Ranzinger S, McCall PJ, Kroeger A, Horstick O. Dengue disease surveillance: an updated systematic literature review. Trop Med Int Health. 2014;19: 1116–1160. 10.1111/tmi.12333 24889501 PMC4253126

[11] Beatty ME, Stone A, Fitzsimons DW, Hanna JN, Lam SK, Vong S, et al Best practices in dengue surveillance: a report from the Asia-Pacific and Americas dengue prevention boards. PLoS Negl Trop Dis. 2010;4: e890–e890. 10.1371/journal.pntd.0000890.t002 21103381 PMC2982842

[12] Ellis AM, Garcia AJ, Focks D, Morrison AC, Scott TW. Parameterization and sensitivity analysis of a complex simulation model for mosquito population dynamics, dengue transmission, and their control. Am J Trop Med Hyg. 2011;85: 257–264. 10.4269/ajtmh.2011.10-0516 21813844 PMC3144822

[13] Racloz V, Ramsey R, Tong S, Hu W. Surveillance of dengue fever virus: a review of epidemiological models and early warning systems. PLoS Negl Trop Dis. 2012;6: e1648 10.1371/journal.pntd.0001648.t001 22629476 PMC3358322

[14] Rigau-Pérez JG, Millard PS, Walker DR, Deseda CC, Casta-Velez A. A deviation bar chart for detecting dengue outbreaks in Puerto Rico. Am J Pub Health. 1999;89: 374–378. 10.2105/AJPH.89.3.374 10076488 PMC1508601

[15] Barbazan P, Yoksan S, Gonzalez J-P. Dengue hemorrhagic fever epidemiology in Thailand: description and forecasting of epidemics. Microbes Infect. 2002;4: 699–705. 10.1016/S1286-4579(02)01589-7 12067829

[16] Thai KTD, Anders KL. The role of climate variability and change in the transmission dynamics and geographic distribution of dengue. Exp Bio Med. 2011;236: 944–954. 10.1258/ebm.2011.010402 21737578

[17] Phung D, Huang C, Rutherford S, Chu C, Wang X, Nguyen M, et al Act Trop. 2015;141: 88–96. 10.1016/j.actatropica.2014.10.005 25447266

[18] Hii YL, Zhu H, Ng N, Ng LC, Rocklöv J. Forecast of dengue incidence using temperature and rainfall. PLoS Negl Trop Dis. 2012;6: e1908 10.1371/journal.pntd.0001908.g004 23209852 PMC3510154

[19] Hii YL, Rocklöv J, Wall S, Ng LC, Tang CS, Ng N. Optimal lead time for dengue forecast. PLoS Negl Trop Dis. 2012;6: e1848 10.1371/journal.pntd.0001848 23110242 PMC3475667

[20] Hii YL, Rocklöv J, Ng N, Tang CS, Pang FY, Sauerborn R. Climate variability and increase in intensity and magnitude of dengue incidence in Singapore. Glob Health Act. 2009;2 10.3402/gha.v2i0.2036 PMC2799326 20052380

[21] Xu H-Y, Fu X, Lee LKH, Ma S, Goh KT, Wong J, et al Statistical modeling reveals the effect of absolute humidity on dengue in Singapore. PLoS Negl Trop Dis. 2014;8: e2805 10.1371/journal.pntd.0002805.t002 24786517 PMC4006725

[22] Campbell KM, Lin CD, Iamsirithaworn S, Scott TW. The complex relationship between weather and dengue virus transmission in Thailand. Am J Trop Med Hyg. 2013;89: 1066–1080. 10.4269/ajtmh.13-0321 23958906 PMC3854883

[23] Favier C, Dégallier N, Dubois MA. Dengue epidemic modeling: stakes and pitfalls. Asia Pacific Biotech. 2005;9: 1191–1194.

[24] Ninphanomchai S, Chansang C, Hii Y, Rocklöv J, Kittayapong P. Predictiveness of disease risk in a global outreach tourist setting in Thailand using meteorological data and vector-borne disease incidences. Int J Env Res Pub Health. 2014;11: 10694–10709. 10.3390/ijerph111010694 25325356 PMC4211001

[25] Johansson MA, Dominici F, Glass GE. Local and global effects of climate on dengue transmission in Puerto Rico. PLoS Negl Trop Dis. 2009;3: e382 10.1371/journal.pntd.0000382.g003 19221592 PMC2637540

[26] Shewhart WA. Economic Control of Quality of Manufactured Product. Bell Sys Tech J. 1931;9: 364–389.

[27] Reid RD, Sanders NR. Operations management: an integrated approach 2nd ed. Management Science; 2004.

[28] Stroup DF, Williamson GD, Herndon JL, Karon JM. Detection of aberrations in the occurrence of notifiable diseases surveillance data. Stat Med. 1989;8: 323–332. 2540519 10.1002/sim.4780080312

[29] Cullen JR, Chitprarop U, Doberstyn EB, Sombatwattanangkul K. An epidemiological early warning system for malaria control in northern Thailand. Bull World Health Organ. 1984;62: 107–114. PMC2536271 6609015

[30] Runge-Ranzinger S, Horstick O, Marx M, Kroeger A. What does dengue disease surveillance contribute to predicting and detecting outbreaks and describing trends? Trop Med Int Health. 2008;13: 1022–1041. 10.1111/j.1365-3156.2008.02112.x 18768080

[31] Kuhn K, Campbell-Lendrum D, Haines A, Cox J, Corvalán C, Anker M, et al Using climate to predict infectious disease epidemics Geneva: WHO 2005.

[32] Stoddard ST, Morrison AC, Vazquez-Prokopec GM, Paz Soldan V, Kochel TJ, Kitron U, et al The role of human movement in the transmission of vector-borne pathogens. PLoS Negl Trop Dis. 2009;3: e481 10.1371/journal.pntd.0000481.t001 19621090 PMC2710008

[33] Stoddard ST, Forshey BM, Morrison AC, Paz-Soldan VA, Vazquez-Prokopec GM, Astete H, et al House-to-house human movement drives dengue virus transmission. Proc Natl Acad Sci USA. 2013;110: 994–999. 10.1073/pnas.1213349110 23277539 PMC3549073

[34] Harrington J, Kroeger A, Runge-Ranzinger S, O'Dempsey T. Detecting and responding to a dengue outbreak: evaluation of existing strategies in country outbreak response planning. J Trop Med. 2013: 1–9. 10.1111/j.1365-3156.2010.02489.x PMC3810135 24222774

[35] Wunderground. The Weather Channel, LLC. Available: http://www.wunderground.com. Accessed 01.09.2014.

[36] TuTiempo. Tutiempo Network, S.L. Available: http://en.tutiempo.net. Accessed 01.09.2014.

[37] Farrington P, Andrews N. Outbreak detection: application to infectious disease surveillance In: Brookmeyer Ron and Stroup Donna F. eds. Monitoring the health of populations: statistical principles and methods for public health surveillance. Oxford University Press, New York; 2003 pp. 203–231.

[38] Chang FS, Tseng YT, Hsu PS, Chen CD, Lian IB, Chao DY. Re-assess vector indices threshold as an early warning tool for predicting dengue epidemic in a dengue non-endemic country. PLoS Negl Trop Dis. 2015;9: e0004043 10.1371/journal.pntd.0004043.s004 26366874 PMC4569482

[39] Chan M, Johansson MA. The incubation periods of dengue viruses. PLoS One. 2012;7: e50972 10.1371/journal.pone.0050972.s003 23226436 PMC3511440

[40] Watts DM, Burke DS, Harrison BA, Whitmire RE, Nisalak A. Effect of temperature on the vector efficiency of Aedes aegypti for dengue 2 virus. Am J Trop Med Hyg. 1987;36: 143–152. 3812879 10.4269/ajtmh.1987.36.143

[41] De Simone TS, Nogueira RMR, Araújo ESM, Guimarães FR, Santos FB, Schatzmayr HG, et al Dengue virus surveillance: the co-circulation of DENV-1, DENV-2 and DENV-3 in the State of Rio de Janeiro, Brazil. T Roy Soc Trop Med H. 2004;98: 553–562. 10.1016/j.trstmh.2003.09.003 15251405

[42] Kroeger A, McCall PJ, Ranzinger SR, Brady OJ, Olliaro P, Petzold M, et al. WHO-TDR Meeting. WHO-TDR. Liverpool; 2014.

[43] STATA 13.1. 13 ed. TX: Stata Corporation. Stata Statistical Software. StataCorp. 2013.

[44] Semenza J. Prototype Early Warning Systems for Vector-Borne Diseases in Europe. IJERPH. 2015;12: 6333–6351. 10.3390/ijerph120606333 26042370 PMC4483704

[45] Halide H, Ridd P. A predictive model for dengue hemorrhagic fever epidemics. Int J Env Health Res. 2008;18: 253–265. 10.1080/09603120801966043 18668414

[46] Lowe DR, Barcellos C, Coelho CAS, Bailey PTC, Coelho GE, Graham R, et al Dengue outlook for the World Cup in Brazil: an early warning model framework driven by real-time seasonal climate forecasts. Lancet Infect Dis. 2014;14: 619–626. 10.1016/S1473-3099(14)70781-9 24841859

[47] Brady OJ, Smith DL, Scott TW, Hay SI. Dengue disease outbreak definitions are implicitly variable. Epidemics. 2015;11: 92–102. 10.1016/j.epidem.2015.03.002 25979287 PMC4429239

[48] Scott TW, Morrison AC. Vector dynamics and transmission of dengue virus: implications for dengue surveillance and prevention strategies vector dynamics and dengue prevention. Curr Top Microbiol. 2010;338: 115–128. 10.1007/978-3-642-02215-9_9 19802582

[49] Karl S, Halder N, Kelso JK, Ritchie SA, Milne GJ. A spatial simulation model for dengue virus infection in urban areas. 2014;14: 1–17. 10.1186/1471-2334-14-447 PMC4152583 25139524

[50] Musso D, Van Mai Cao-Lormeau, Gubler DJ. Correspondence. Lancet. 2015;386: 243–244. 10.1016/S0140-6736(15)61273-9 26194519

[51] Cardona-Ospina JA, Diaz-Quijano FA, Rodríguez-Morales AJ. Burden of chikungunya in Latin American countries: estimates of disability-adjusted life-years (DALY) lost in the 2014 epidemic. Int J Inf Dis. 2015;38: 60–61. 10.1016/j.ijid.2015.07.015 26216764

[52] Guzman MG, Halstead SB, Artsob H, Buchy P, Farrar J, Gubler DJ, et al Dengue: a continuing global threat. Nature. 2010;8: S7–S16. 10.1038/nrmicro2460 PMC4333201 21079655

[53] Reiner RC, Stoddard ST, Forshey BM, King AA, Ellis AM, Lloyd AL, et al PNAS plus: from the cover: time-varying, serotype-specific force of infection of dengue virus. Proc Natl Acad Sci. 2014;111: e2694–2702. 24847073 10.1073/pnas.1314933111 PMC4084484

[54] Morrison AC, Minnick SL, Rocha C, Forshey BM, Stoddard ST, Getis A, et al Epidemiology of dengue virus in Iquitos, Peru 1999 to 2005: Interepidemic and epidemic patterns of transmission. PLoS Negl Trop Dis. 2010;4: e670 10.1371/journal.pntd.0000670.t008 20454609 PMC2864256

[55] Rodrigues HS, Monteiro MTT, Torres DFM, Zinober A. Dengue disease, basic reproduction number and control. Int J Comp Math. 2012;3: 334–346. http://dxdoiorg/101080/002071602011554540.

[56] Nisalak A, Endy TP, Nimmannitya S, Kalayanarooj S, Thisayakorn U, Scott RM, et al Serotype-specific dengue virus circulation and dengue disease in Bangkok, Thailand from 1973 to 1999. Am J Trop Med Hyg. 2003;68: 191–202. 12641411

[57] Stoddard ST, Wearing HJ, Reiner RC, Morrison AC, Astete H, Vilcarromero S, et al Long-term and seasonal dynamics of dengue in Iquitos, Peru. PLoS Negl Trop Dis. 2014;8: e3003 10.1371/journal.pntd.0003003.s028 25033412 PMC4102451

[58] Christiansen-Jucht CL, Parham PE, Saddler A, Koella JC, Basanez M-G. Temperature during larval development and adult maintenance influences the survival of Anopheles gambiae s.s. 2014;7: 1–10. 10.1186/s13071-014-0489-3 PMC4236470 25367091

[59] Yi B, Zhang Z, Xu D, Xi Y. Relationship of dengue fever epidemic to Aedes density changed by climate factors in Guangdong Province. J Hyg Res. 2003;32: 152–154. 12793011

[60] Zhang Y, Bi P, Hiller JE. Climate change and the transmission of vector-borne diseases: a review. Asia-Pacific J Pub Health 2008;20: 64–76. 10.1177/1010539507308385 19124300

[61] Pham HV, Doan HTM, Phan TTT, Minh NNT. Ecological factors associated with dengue fever in a central highlands province, Vietnam. Stoch Env Res Risk Assess. 2011;25: 485–494. 10.1007/s00477-010-0417-9 PMC3126728 21679398

[62] Rabaa MA, Simmons CP, Fox A, Le MQ, Nguyen TTT, Le HY, et al Dengue virus in sub-tropical northern and central Vietnam: population immunity and climate shape patterns of viral invasion and maintenance. PLoS Negl Trop Dis. 2013;7: e2581 10.1371/journal.pntd.0002581.s003 24340118 PMC3854975

[63] Tun-Lin W, Burkot TR, Kay BH. Effects of temperature and larval diet on development rates and survival of the dengue vector Aedes aegypti in North Queensland, Australia. Med Vet Ent. 2000;14: 31–37. 10.1046/j.1365-2915.2000.00207.x 10759309

[64] Hugo LE, Jeffery JAL, Trewin BJ, Wockner LF, Thi Yen N, Le NH, et al Adult survivorship of the dengue mosquito Aedes aegypti varies seasonally in in Central Vietnam. PLoS Negl Trop Dis. 2014;8: e2669 10.1371/journal.pntd.0002669 24551251 PMC3923839

[65] Meza-Ballesta A, Gónima L. The influence of climate and vegetation cover on the occurrence of dengue cases (2001–2010). Rev Sal Pub (Bogota, Colombia). 2014;16: 293–306. 25383502

[66] Tran HP, Adams J, Jeffery JAL, Nguyen YT, Vu NS, Kutcher SC, et al Householder perspectives and preferences on water storage and use, with reference to dengue, in the Mekong Delta, southern Vietnam. Roy Soc Trop Med Hyg; 2010;2: 136–142. 10.1016/j.inhe.2009.12.007 24037472

[67] Betts RA, Cox PM, Collins M, Harris PP, Huntingford C, Jones CD. The role of ecosystem-atmosphere interactions in simulated Amazonian precipitation decrease and forest dieback under global climate warming. Theor Appl Climatol. 2004;78 10.1007/s00704-004-0050-y

[68] Morin CW, Comrie AC, Ernst K. Climate and dengue transmission: evidence and implications. Env Health Persp. 2013;121: 1264–1272. 10.1289/ehp.1306556 PMC3855512 24058050

[69] Naish S, Dale P, Mackenzie JS, McBride J, Mengersen K, Tong S. Climate change and dengue: a critical and systematic review of quantitative modelling approaches. BMC Infect Dis. 2014;14: 1–14. 10.1186/1471-2334-14-167 24669859 PMC3986908

[70] Chen S-C, Hsieh M-H. Science of the total environment. 2012;431: 385–391. 10.1016/j.scitotenv.2012.05.012 22705874

[71] Johansson MA, Cummings DAT, Glass GE. Multiyear climate variability and dengue—el niño southern oscillation, weather, and dengue incidence in Puerto Rico, Mexico, and Thailand: a longitudinal data analysis. PLoS Med. 2009;6: e1000168 10.1371/journal.pmed.1000168.s006 19918363 PMC2771282

[72] Lee KS, Yee-Ling L, Sharon L, Barkham T, Aw P, Peng-Lim Ooi, et al Dengue Virus Surveillance for Early Warning, Singapore. Emer Infect Dis. 2010 5 16. 10.3201/eid1605.091006 PMC2953985 20409381

[73] Yamanaka A, Mulyatno KC, Susilowati H, Hendrianto E, Ginting AP. Displacement of the predominant dengue virus from type 2 to type 1 with a subsequent genotype shift from IV to I in Surabaya, Indonesia 2008–2010. PLoS ONE. 2011:6; e27322 10.1371/journal.pone.0027322 22087290 PMC3210158

[74] Chan M, Johansson MA. The incubation periods of dengue viruses. PLoS ONE. 2012;7: e50972 10.1371/journal.pone.0050972.s003 23226436 PMC3511440

[75] Rosenbaum L. Communicating uncertainty—ebola, public health, and the scientific process. N Engl J Med. 2015;372: 7–9. 10.1056/NEJMp1413816 25394322

[76] Chan EH, Sahai V, Conrad C, Brownstein JS. Using web search query data to monitor dengue epidemics: a new model for neglected tropical disease surveillance. PLoS Negl Trop Dis. 2011;5: e1206 10.1371/journal.pntd.0001206.t001 21647308 PMC3104029

[77] Althouse BM, Ng YY, Cummings DAT. Prediction of dengue incidence using search query surveillance. PLoS Negl Trop Dis. 2011;5: e1258 10.1371/journal.pntd.0001258.s001 21829744 PMC3149016

[78] Chunara R, Andrews JR, Brownstein JS. Social and news media enable estimation of epidemiological patterns early in the 2010 Haitian cholera outbreak. Am J Trop Med Hyg. 2012;86: 39–45. 10.4269/ajtmh.2012.11-0597 22232449 PMC3247107

[79] Liu-Helmersson J, Stenlund H, Wilder-Smith A, Rocklöv J. Vectorial capacity of Aedes aegypti: effects of temperature and implications for global dengue epidemic potential. PLoS ONE. 2014;9: e89783 10.1371/journal.pone.0089783.s002 24603439 PMC3946027

[80] Hernández-Ávila JE, Rodríguez M-H, Santos-Luna R, Sánchez-Castañeda V, Román-Pérez S, Ríos-Salgado VH, et al Nation-wide, web-based, geographic information system for the integrated surveillance and control of dengue fever in Mexico. PLoS ONE. 2013;8: e70231 10.1371/journal.pone.0070231.s001 23936394 PMC3735575

[81] Louis VRR, Phalkey R, Horstick O, Ratanawong P, Wilder-Smith A, Tozan Y, et al Modeling tools for dengue risk mapping—a systematic review. 2014;13: 1–15. 10.1186/1476-072X-13-50 PMC4273492 25487167

[82] Bowman LR, Runge-Ranzinger S, McCall PJ. Assessing the relationship between vector indices and dengue transmission: a systematic review of the evidence. PLoS Negl Trop Dis. 2014;8: e2848 10.1371/journal.pntd.0002848.s003 24810901 PMC4014441

[83] Rueda LM, Patel KJ, Axtell RC, Stinner RE. Temperature-dependent development and survival rates of Culex quinquefasciatus and Aedes aegypti (Diptera: Culicidae). J Med Entomol. 1990;27: 892–898. 2231624 10.1093/jmedent/27.5.892

[84] Shi Y, Liu X, Kok S-Y, Rajarethinam J, Liang S, Yap G, et al Three-month real-time dengue forecast models: An early warning system for outbreak alerts and policy decision support in Singapore. Env Health Per. 2015; [Epub ahead of print]. 10.1289/ehp.1509981 PMC5010413 26662617

[85] Bowman LR, Donegan S, McCall PJ. Is dengue vector control deficient in effectiveness or evidence?: Systematic review and meta-analysis. PLoS Negl Trop Dis. 2016;10: e0004551 10.1371/journal.pntd.0004551.s012 26986468 PMC4795802

